# Lipid metabolism is dysregulated in a mouse model of diabetes

**DOI:** 10.1007/s11306-022-01884-w

**Published:** 2022-05-31

**Authors:** Samuel Furse

**Affiliations:** 1 Core Metabolomics and Lipidomics Laboratory, Wellcome-MRC Institute of Metabolic Science, University of Cambridge, Addenbrooke’s Treatment Centre, Keith Day Road Cambridge, Cambridge, CB2 0QQ UK; 2 Metabolic Disease Unit, Wellcome-MRC Institute of Metabolic Science, University of Cambridge, Addenbrooke’s Treatment Centre, Keith Day Road Cambridge, Cambridge, CB2 0QQ UK; 3 Biological Chemistry Group, Jodrell Laboratory, Royal Botanic Gardens Kew, Richmond, TW9 3SD UK

**Keywords:** Lipid traffic analysis, T2DM, Lipid metabolism

## Abstract

Much evidence for diabetes mellitus being associated with dysregulated lipid metabolism has been accrued from studies using blood plasma. However, the systemic dysregulation these results point to is not understood. This study used Lipid Traffic Analysis on data from a mouse model of diabetes to test the hypothesis that the systemic control of lipid metabolism differed in a model of diabetes. This provided eidence for changes in the systemic control of both triglyceride and phospholipid metabolism that were not attributable to dietary intake. This supports the conclusion that diabetes is a systemic condition associated with dysregulated lipid metabolism through several pathways.

**Supplementary Information:**

The online version contains supplementary material available at 10.1007/s11306-022-01884-w.

## Introduction

Diabetes is increasingly being recognised as serious threat to the health of humans and companion animals. Diabetes is increasingly common across the globe, especially in the over 30s (Foundation, 2021; Wright et al., 2006; Zhao et al., 2017). The condition is increasingly common in pets and occasionally in wild animals and is associated with excess nutrition (Delicano et al., 2020; Heeley et al., 2020). The hyperglycaemia associated with diabetes has been well characterised, however it has not been able to explain the consequences observed, or the increased risk of cardio-vascular disease. This has led to research interest in the molecular mechanisms that may be involved. Research in this area has shown that several metabolic pathways are associated with onset of type 2 diabetes mellitus (T2DM), identified through lipid and amino acid biomarker discovery (Savage et al., 2007; Suvitaival et al., 2018; Wright et al., 2006; Zhao et al., 2017).

Molecular studies of diabetes are of interest because they may be able to tell us which metabolites or pathways are dysregulated in advance of the condition becoming fully established. Although this process has begun in T2DM, it is more advanced in gestational diabetes mellitus (GDM). GDM is also characterised by hyperglycaemia and has similar risk factors to T2DM, *e.g.* obesity. GDM differs strongly in that it occurs only during pregnancy, with hyperglycaemia disappearing quickly after delivery. The shorter onset time of GDM means it has been easier to research and subsequently its molecular origins have received a good deal of attention. Research into the lipid metabolism around GDM has shown that lipid metabolism is disrupted in humans at least 10 weeks before diagnosis of hyperglycaemia (Furse et al., 2019; Lu et al., 2016), that the lipid composition of maternal blood plasma is different before, during and after the onset of GDM (Furse et al., 2021a) and that dysregulated lipid metabolism persists throughout the maternal system after weaning (Furse et al., 2022a). This evidence strongly suggests that dysregulated lipid metabolism has a role in the pathophysiology of GDM, raising questions about a possible role for dysregulated lipid metabolism in T2DM.

The evidence from studies of GDM that shows dysregulation of lipid metabolism in advance of hyperglycaemia becoming established can be used to inform interpretation of lipid metabolism in T2DM. For example, FAs associated with de novo lipogenesis are negatively associated with insulin sensitivity (Johnston et al., 2018) and lipid oxidation is positively associated with T2DM (Kramer et al., 2019), suggesting that regulation of both lipid synthesis and oxidation may be linked to diabetes. Chen et al*.* found that lipid metabolism and oxidation were disrupted in organs known to be involved in diabetes, using a comprehensive set of tissue-tissue comparisons (Chen et al., 2020). This has revealed several local effects. However, importantly, both lipid metabolism and T2DM are known to be system-wide and affect several inter-linked pathways. Though useful for identification of biomarker discovery, tissue-tissue comparisons do not represent a system-level analysis. We therefore tested the hypothesis that systemic dysregulation of lipid metabolism was associated with diabetes using a network analysis tool capable of revealing long-range effects (Chen et al., 2020), in silico.

Lipid Traffic Analysis (LTA) is the ideal tool for answering this question as it uses differences in the spatial distribution of metabolites, between control and experimental groups, to identify how and where control mechanisms differ between systems (Furse et al., 2021a, b; 2022a, b). We applied LTA to the published lipidomics data, fitted into the network shown in Fig. S1. This adds considerably to simple tissue-tissue comparisons as it can identify changes throughout a system, as well as any that exist through more than one organ. For example, the effects of changes to de novo lipogenesis in the liver also affect which and how much triglyceride arrive at the heart. This system-level analysis therefore offers insight into diabetes that cannot be achieved any other way.

## Materials and methods

### Animal model and lipidomics data

Lipidomics data for this study were publicly available from an Open Access paper(Chen et al., 2020) and collected using LCMS and MS/MS(Chen et al., 2020; Wu et al., 2019) with in-house developed standards. The mouse model used for the collection has been described in publicly available reports and is based upon two phenotypes, namely a control group and a diabetic group (high-fat-fed and treated with streptozotocin Chen et al., 2020; Yu et al., 2017). Up to 220 lipid variables were identified in liver, brain, heart atria, heart ventricles, spleen, renal medulla and renal cortex homogenates and in serum.

### Lipid traffic analysis

Lipid Traffic Analysis v2.3 was used for this study (Furse et al., 2021a; 2022a, b). The code was executed in RStudio(v1.2.5x). The full code for Lipid Traffic Analysis v2.3 used in this study can be found in the *Supplementary Information file 1*. The tissues were mapped to a known biological/metabolic network (Fig. S1). LTA calculated error-normalised fold change and categorised lipids for the Switch Analysis according to whether they were ***A***, ***B*** or ***U***-type lipids. Jaccard-Tanimoto Coefficients (JTCs, *J*) and associated *p* values were used as a non-parametric measure of the distinctions between lipid variables associated with phenotype(s). The *p* value associated with each *J* represents the probability that the difference between the lists of variables for the two phenotypes occurred by random chance. It represents both the number of variables only found in either of the two groups and the order of the binary list. Variables were regarded as present if they had a signal strength > 0 in ≥ 66% of samples per group.

### Statistical methods

Calculations were done in Microsoft Excel 2016. Graphs were prepared in Excel 2016 or OriginLab 2018.

## Results and discussion

**Fig. 1:**
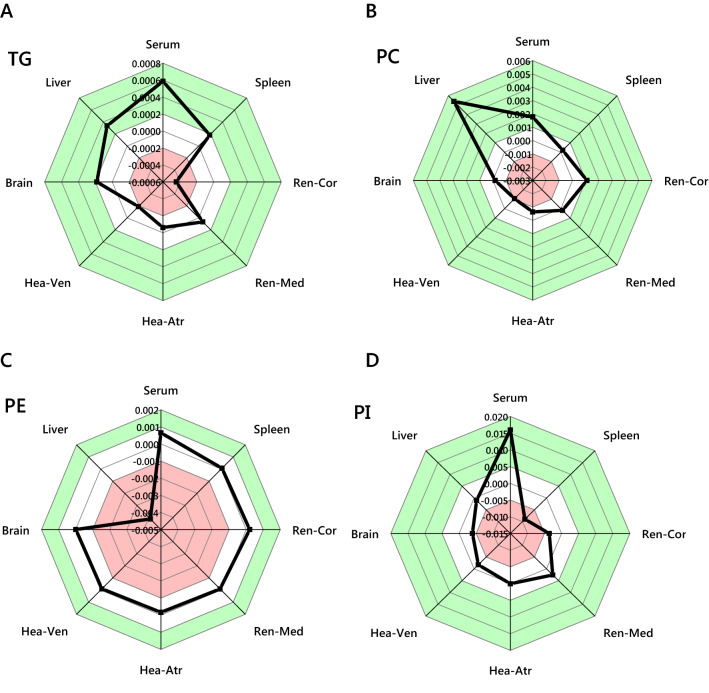
The abundance of lipid classes across tissue types, expressed as error normalised fold change (ENFC (Furse et al., 2021b)) between control and diabetic mice. Panel **A** Triglycerides (TGs); **B** Phosphatidylcholines (PCs); **C** Phosphatidylethanolamines; **D** Phosphatidylinositols. The green areas represent an increase in the abundance of the respective lipid head group of at least one division above 0 in the diabetic group, whereas the red zone represents a decrease in abundance of that lipid head group with respect to the control group

**Fig. 2:**
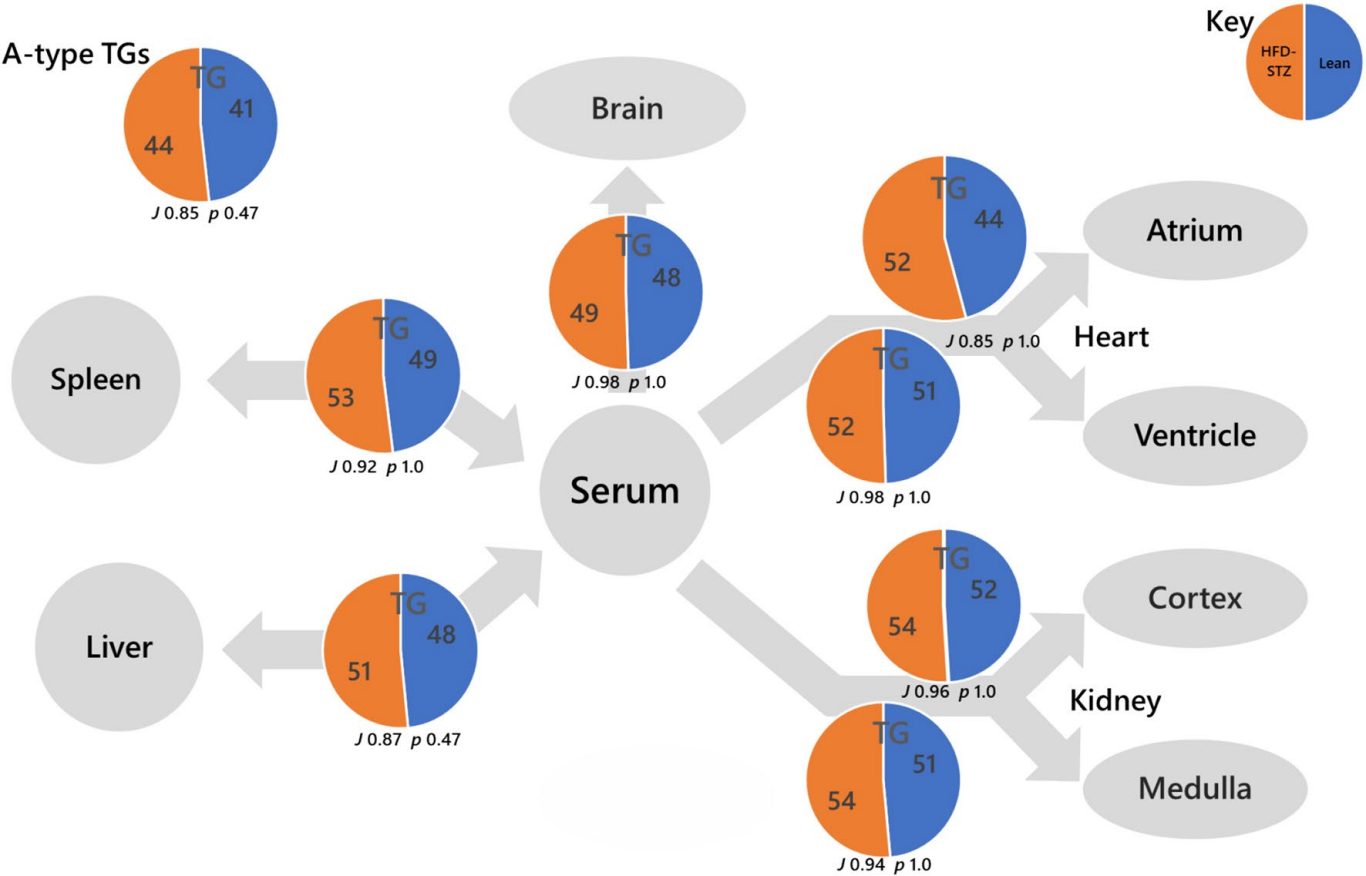
Switch analysis of triglyceride (TG) variables in a mouse model of diabetes. Inset pie chart shows the ***A***-type TGs. No ***U***-type lipids were detected. The Jaccard-Tanimoto coefficients (*J*) and probability (*p*) values that describe the similarity between sets of variables

The systemic examination of the lipidomics data from a model of diabetes used in the present study (Chen et al., 2020) began with an investigation of lipid pathways. An Abundance Analysis of lipid head groups (classes) showed that triglycerides (TGs), phosphatidylcholines (PCs), phosphatidylethanolamines (PEs) and phosphatidylinositols (PIs) were all altered in this network (Fig. 1). This showed that the systemic controls of lipid metabolism differed in the diabetic (HFD-STZ) phenotype through different changes in individual pathways. These results suggest that several lipids may therefore be involved in T2DM and so an untargeted analysis was used to plot the spatial distribution of all lipids measured. This is known as Switch Analysis and shows the difference in lipid distribution between the two phenotypes through the whole biological network (Fig. S1). The Switch Analysis identified several lipids as ubiquitous in either one of the two phenotypes (Fig. S2) and also pinpointed differences in distribution of all lipid isoforms for the four most populous lipid head groups (TG, PC, PE and PI, Figs. 2, S3–4). These lipid classes were therefore investigated more closely.

The Switch Analysis of TGs showed that the profile of TGs differed consistently between the two groups throughout the system but also that the phenotypes differed specifically between the Liver-Serum axes of the two groups (Fig. 2). The variables that distinguished the two systems were TG(44:0, 46:0) in the control group and TG(52:7, 58:11, 60:10, 60:11, 60:12) in the diabetic group. These variables are associated with de novo lipogenesis (DNL, Sanders et al., 2018) and the dietary intake of PUFAs, respectively. As these two distinct subtypes of TG differed between phenotypes, we undertook a quantitative analysis of TG subclasses. An abundance analysis of established markers for DNL showed that they were typically less abundant in the livers of diabetic mice than controls, and if not then lower in abundance than it was in either brain or serum (Fig. S5). By contrast, the livers of diabetic mice were generally more abundant in PUFA-TGs than controls, as was serum (Fig. S6). However, the abundance of the same species was lower in the spleens of diabetic mice. This also showed that how the distribution of TGs is controlled differs in this model of diabetes. Furthermore, a difference in the control of lipid metabolism in T2DM is consistent with a model of GDM, which also showed a change in how TG metabolism involving the liver differs between in mice who experienced diabetes in pregnancy (Furse et al., 2022a).

The Switch Analysis of the PI variables showed that there are more PI variables in the diabetic mice and also that the Serum-Brain axis differs between the two phenotypes (Fig. S4). This showed that the control of PI metabolism in the CNS as well as the periphery differed in this model of diabetes. The isoforms by which the two groups differ are structural PIs rather than signalling ones; PI(36:2, 36:3) appear on the Serum-Brain axis of the control group but not that of the diabetic group whereas PI(36:1, 38:6) were found on this axis of the diabetic group but not the control group. This showed that there was a shift in the distribution of PIs in the CNS in diabetes, away from C_36_ isoforms, suggesting that the physical behaviour in membranes may be altered. This was an unexpected result as presently there is little evidence for changes to the CNS in T2DM. This led us to do an abundance analysis of other important isoforms of PI throughout the network (Fig. S7). This showed that there was very little difference in PIs in heart tissues between groups, with the greatest differences observed in serum, showing that C_34_ and C_36_ isoforms were more abundant in this compartment. Importantly, PI(32:1), an isoform of PI closely associated with structural roles and not at all associated with signalling, was much lower in the serum of diabetic mice.

The biggest shift across the system in diabetic mice was in PI(38:6), which was lower in all compartments except the liver in which it was higher. Notably, the distribution of polyunsaturated TGs also differed in diabetic mice, however with a different pattern (Fig. S6). Such a difference in the distribution of PUFA-containing isoforms of PI and TG showed that as well as different biosynthesis in several lipid pathways, the distribution of PUFA-containing lipids differs in diabetic systems. This showed that the supply of FAs is altered in this model of diabetes.

Alterations in the distribution of PUFA-containing also noted in PCs and PEs, though differed to changes in PI and TG. The Switch Analysis showed that PC(32:1, 32:2, 34:4, 36:6) and PE(40:7) were found throughout the control network but not in the diabetic one (Fig. S3). This is consistent with the finding described above in which the distribution of chain lengths was shifted in the PI fraction of diabetic mice. It shows that whilst the PI profile broadens in diabetic mice, the PC and PE profile narrows.

The systemic alterations in lipid metabolism found in this model of diabetes are also consistent with changes in lipid metabolism associated with GDM. In these models, systemic changes in the distribution of PUFA-PCs, PUFA-TGs and TGs associated with DNL were observed. In the model of GDM, these pathways are not modulated correctly through pregnancy and the dysregulation persisted after delivery (Furse et al., 2021a; 2022a). Changes in the distribution of DNL-associated TGs also provides evidence for a difference in control between the control and diabetic systems, both because the spatial distribution of lipids changes, but also because the intake of carbohydrate remains constant.

Generally, phospholipids also showed differences in how lipid metabolism is controlled, with the suggestion that the profile of PC and PI are changed in opposite directions. This may mean that the physical properties of the membranes in the two systems are roughly equivalent in the short-term, but that they may respond differently to environmental stresses or have different viability in the long term. This observation is consistent with a recent analysis of a model of gestational diabetes that generated the hypothesis that altered lipid composition of membranes drives insulin resistance through decreased activity of membrane proteins such as the insulin receptor (Furse et al., 2022a).

The present study was based on publicly-available lipidomics data from a model of diabetes based on high BMI and administration of streptozotocin (Chen et al., 2020). This model is a useful model of diabetes, however further work is required to characterise it fully, for example how resistant the system is to insulin. The loss of insulin secretion is clear but secondary effects of streptozotocin are unknown. This model is also focused on diabetes in obesity. Obesity is an important risk factor for diabetes, particularly T2DM and GDM, but is not universal. The context of this model is thus diabetes in obesity with low insulin secretion and reception.

## Conclusions

The results from this study show that the system-wide control of lipid metabolism differs in a mouse model of diabetes. The alterations associated with diabetes in TGs are complicated, but show different control of de novo lipogenesis and availability of PUFA-containing TGs. We therefore conclude that the control of lipid distribution differed between the two groups, specifically through traffic between the liver and serum (TGs) and the periphery and CNS (PIs). Further work is required to establish the precise role of each of the systemic differences in T2DM, however the lipid traffic analysis of this mouse model shows for the first time that altered lipid metabolism with respect to key processes such as de novo lipogenesis and structures such as plasma membranes, are associated with diabetes.

## Supplementary Information

Below is the link to the electronic supplementary material.

Supplementary file1 (TXT 32 kb)

Supplementary file2 (DOCX 1000 kb)

## Data Availability

The R code used in the present study for Lipid Traffic Analysis v2.3 is publicly available (Furse et al., 2021a; 2022a, b). (LTA v1.0 is also available publicly (Furse et al., 2021b)).
