# Supplementary material for: Lipid metabolism is dysregulated in a mouse model of diabetes
Source: Metabolomics. 2022 May 31;18(6):36. doi: 10.1007/s11306-022-01884-w (PMC9156495; doi:10.1007/s11306-022-01884-w)
Supplement: Supplementary file 2 — Supplementary file2 (DOCX 1000 kb) [file 11306_2022_1884_MOESM2_ESM.docx]

**Supplementary Figures and Tables**


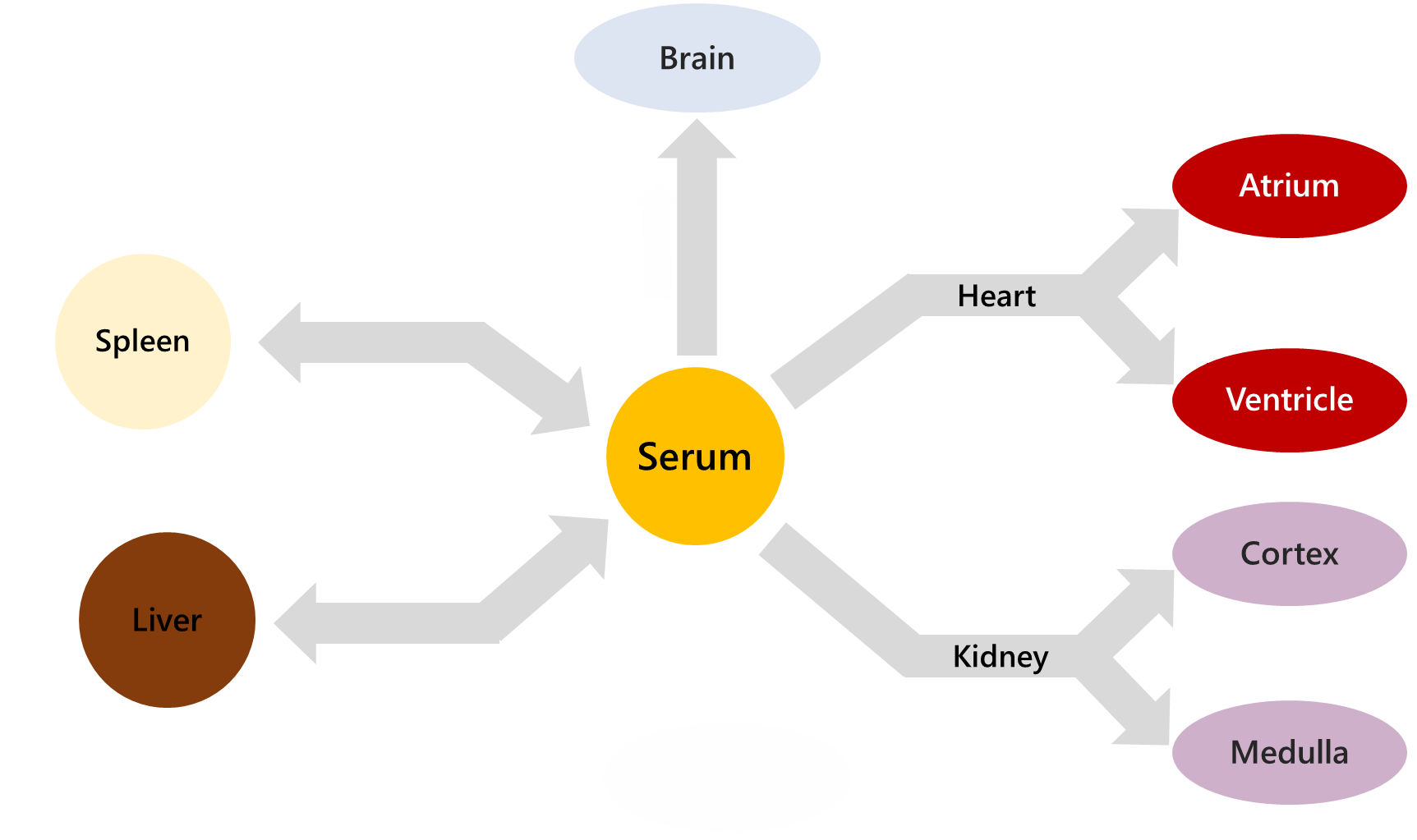


**Fig. S1. The mouse model and tissue network used in the present study.** Schematic representation of the metabolic network that describes the lipid traffic associated with this model. The metabolic relationship between tissues was used as the structure of the network for all analyses in the present study. Lean mice were fed a diet of normal chow *ad libitum* whereas Obese refers to mice fed a high fat diet and streptozotocin([Chen *et al.* 2020](#_ENREF_1)).


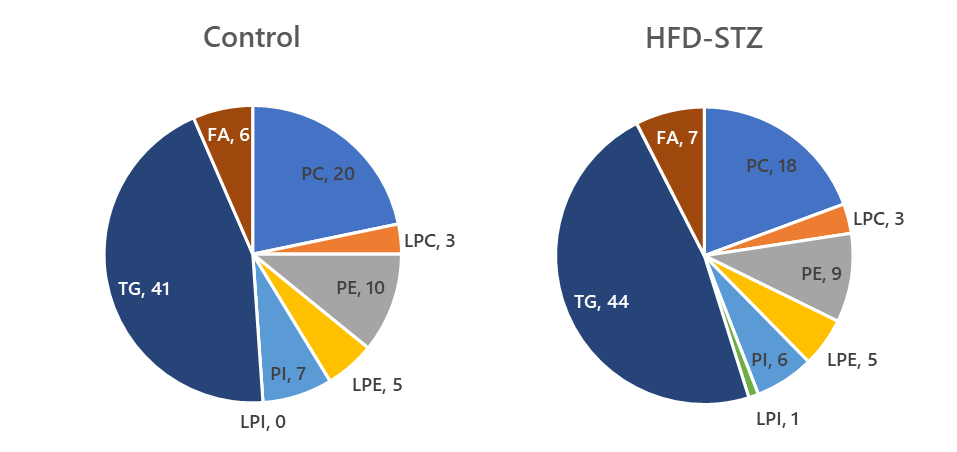

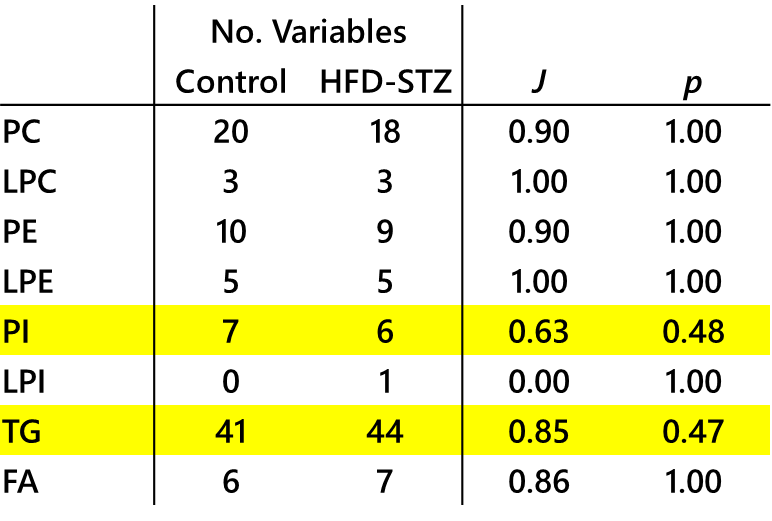


**Fig S2. Variables found trhoughout the control and diabetic phenotypes used in the present study, as identified by a Switch Analysis form an LTA.** The Jaccard-Tanimoto coefficients (*J*) and probability (*p*) values that describe the similarity between sets of variables.

**
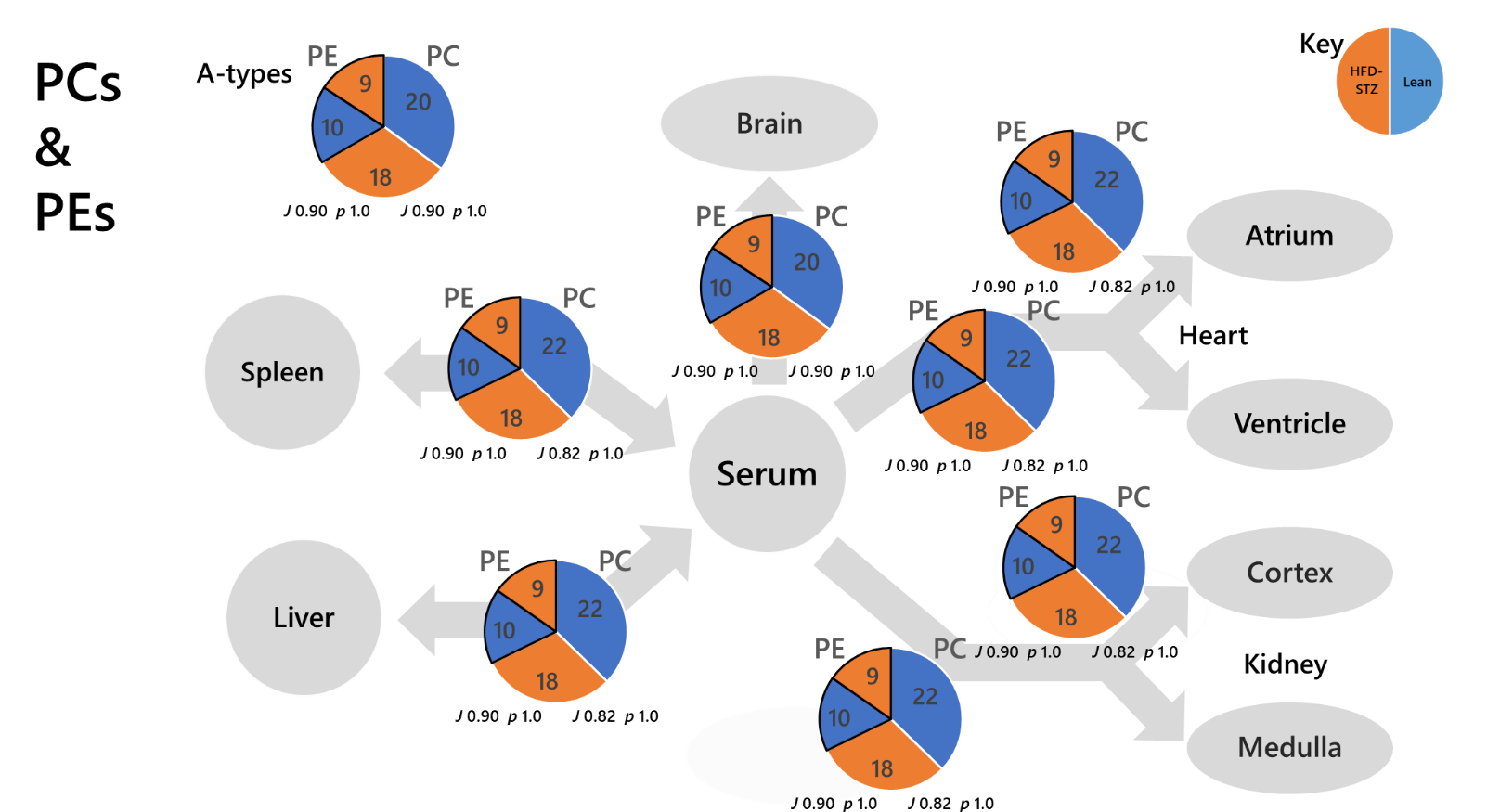
**

**Fig. S3. Switch analysis of phosphatidylcholine (PC) and phosphatidylethanolamine (PE) variables in a mouse model of diabetes.** Inset pie chart shows the ***A***-type variables. The Jaccard-Tanimoto coefficients (*J*) and probability (*p*) values that describe the similarity between sets of variables.


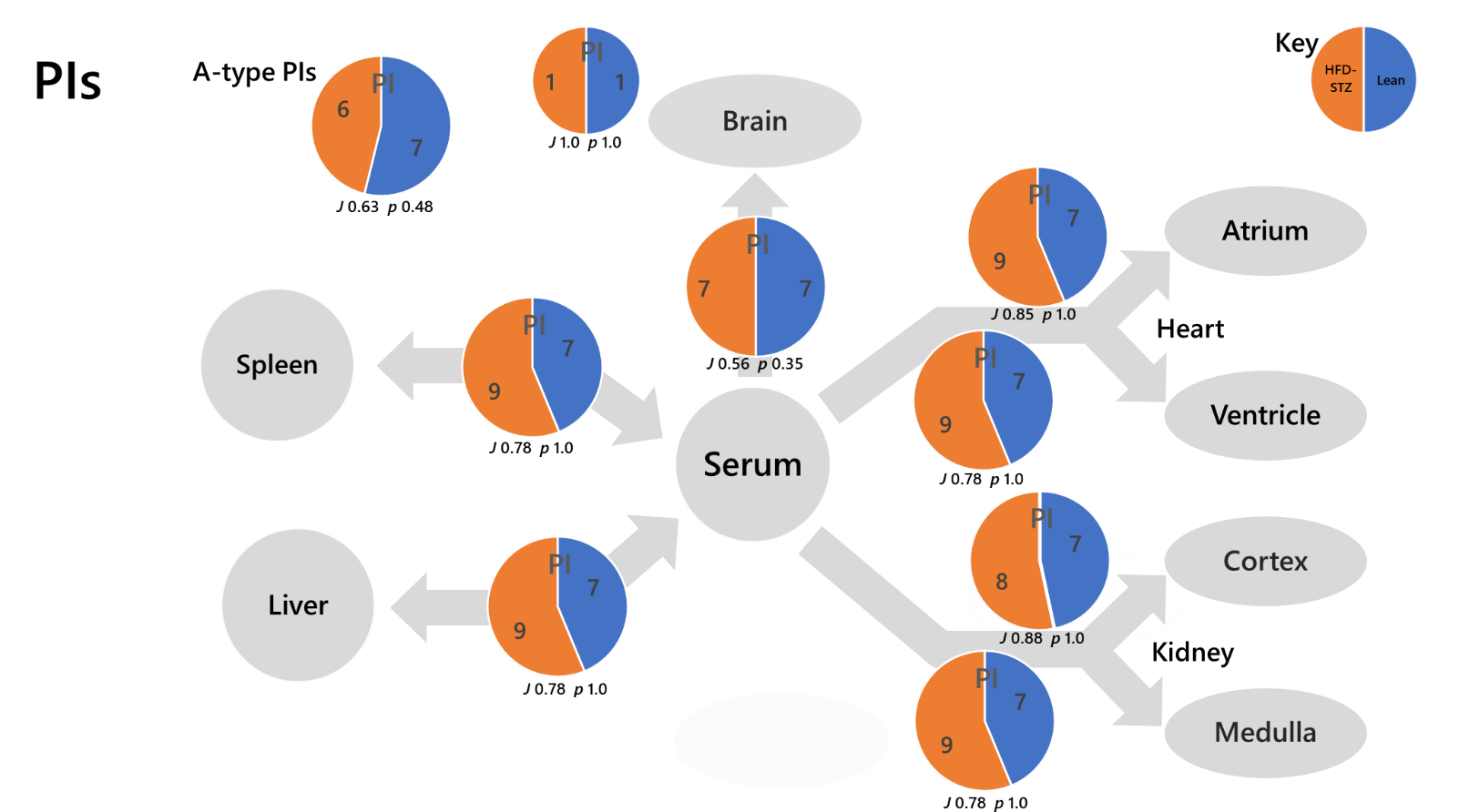


**Fig. S4. Switch analysis of phosphatidylinositol (PI) variables in a mouse model of diabetes.** Inset pie chart shows the ***A***-type TGs. ***U***-type lipids were only detected in the brain tissue. The Jaccard-Tanimoto coefficients (*J*) and probability (*p*) values that describe the similarity between sets of variables.

A B

**** ****

C D

**** ****

**Fig. S5. The abundance of triglyceride markers of *de novo* lipogenesis across tissue types, expressed as error normalised fold change (ENFC(**[**Furse *et al.* 2021b**](#_ENREF_6)**)) between control and diabetic mice**. The green areas represent an increase in the abundance of the respective lipid head group of at least one division above 0 in the diabetic group, whereas the red zone represents a decrease in abundance of that lipid head group with respect to the control group. Hea-Atr, heart atria; Hea-Ven, heart ventricles; Ren-Cor, renal cortex; Ren-Med, renal medulla.

A B

C D

**** ****

E

**Fig. S6. The abundance of polyunsaturated triglycerides across tissue types, expressed as error normalised fold change (ENFC(**[**Furse *et al.* 2021b**](#_ENREF_6)**)) between control and diabetic mice**. The green areas represent an increase in the abundance of the respective lipid head group of at least one division above 0 in the diabetic group, whereas the red zone represents a decrease in abundance of that lipid head group with respect to the control group. Hea-Atr, heart atria; Hea-Ven, heart ventricles; Ren-Cor, renal cortex; Ren-Med, renal medulla.

A B

C D

**** ****

E F

**Fig. S7. The abundance of mono- and polyunsaturated phoshatidylinositols across tissue types, expressed as error normalised fold change (ENFC(**[**Furse *et al.* 2021b**](#_ENREF_6)**)) between control and diabetic mice**. The green areas represent an increase in the abundance of the respective lipid head group of at least one division above 0 in the diabetic group, whereas the red zone represents a decrease in abundance of that lipid head group with respect to the control group. Hea-Atr, heart atria; Hea-Ven, heart ventricles; Ren-Cor, renal cortex; Ren-Med, renal medulla.
